# Convulsive Status Epilepticus Induced by Electroconvulsive Therapy in a Patient with Major Depression

**DOI:** 10.1155/2022/8545991

**Published:** 2022-03-17

**Authors:** Emilie Wieben, Marianne Juel Kjeldsen, Claus H. Sørensen

**Affiliations:** ^1^Odense University Hospital, Department of Psychiatry, Odense, Denmark; ^2^Odense University Hospital, Department of Neurology, Odense, Denmark

## Abstract

Electroconvulsive therapy (ECT) is a well-known, safe, and efficient treatment for a variety of psychiatric diseases. We present here an unusual case of a 34-year-old patient with major depression, who developed convulsive status epilepticus persistent for eight days in connection to her first ECT—a very uncommon but serious complication. The patient was, prior to ECT treatment, treated with lithium carbonate and clomipramine for her depression. Six years prior to the ECT, the patient had experienced a convulsive syncope resulting in traumatic subarachnoid haemorrhage. This case emphasizes the importance of medical recording to detect possible risk factors when considering ECT treatment.

## 1. Introduction

Electroconvulsive therapy (ECT) is an efficient treatment with rapid effects for severe serious psychiatric conditions such as catatonia, schizophrenia, and mood disorders [1, 2].

Seizures provoked by ECT increase the seizure threshold and reduce the probability of seizure. Therefore, ECT is a potential therapeutic strategy for refractory status epilepticus (SE) [3], which is considered safe under general anaesthesia [1, 4]. Side effects and complications can occur such as headache, muscle pain, aspiration, cardiovascular complications, postictal confusion, anterograde and retrograde amnesia, prolonged seizure, and rarely SE [1]. SE is defined as sustained or repeated epileptic seizure that lasts for more than 30 minutes and can be divided into status epilepticus with and without convulsions. Over the last couple of decades, only 13 cases of generalized nonconvulsive SE [5] and three cases of generalized convulsive SE provoked by ECT [6–8] have been identified in the literature. SE is associated with more than 20% mortality among adults [1, 9]. As far as we know, this is the first case of a patient who developed sustained convulsive SE persistent for eight days following the first session of ECT.

## 2. Case Presentation

A 34-year-old female patient known with major depression was admitted voluntarily to an inpatient psychiatric unit due to a severe depressive episode in June 2017. She presented with sadness, loss of interest, lack of energy, feeling hopelessness, decreased concentration, insomnia, excessive guilt, and low self-esteem. The patient showed depressed mood, reduced mimics, psychomotor retardation, and minimal eye contact. Her medical history was unremarkable, with no history of alcohol or illegal drug usage. Physical examination showed no neurological symptoms. The patient's psychiatric medical history was first described in 2002 where she was treated with clomipramine and psychotherapy in an outpatient psychiatric clinic. She was admitted to a psychiatric hospital with depressive symptoms for the first time in 2007 and again in 2014 and 2016. Between the admissions, she was treated in an outpatient psychiatric clinic.

Regarding her somatic medical history, she had a history of one episode of convulsive syncope following a hepatitis vaccine in 2011. Unfortunately, the patient suffered a minor traumatic subarachnoid haemorrhage secondary to her syncope. There was no need for further intervention for the haemorrhage, and the patient did not have any recorded sequelae. She had no family history of epilepsy.

Back in 2017 at her admission to the psychiatric hospital, the patient's depression continued to worsen over the first month of the hospitalization. Lithium carbonate 300 mg/day was added as an antidepressant agent according to the national treatment guidelines, but with no significant improvement in her depression. Serum clomipramine was 1188 nmol/L, and serum lithium was 0.8 mmol/L. She revealed recurrent thoughts of death and suicidal ideation. The patient did not respond to the treatment, with a further progression in her depression. Therefore, a course of ECT was recommended according to national treatment guidelines. Informed consent, regarding ECT treatment, was obtained from both the patient and her family.

Electrocardiography and routine blood tests—including haemoglobin, leukocytes, liver function test, creatinine, electrolytes, and thyroid-stimulating hormone—were all normal. Neither lithium carbonate nor clomipramine was discontinued prior to ECT.

The anaesthetic agents consisted of 175 mg thiopentone followed by 30 mg of succinylcholine intravenously. She was preoxygenated with 100% oxygen, just before electrical stimulation. ECT was administrated, according to the unit's protocol, with Thymatron® apparatus DG, with bilateral frontotemporal placed electrodes with an electrical stimulus of 25% (126 millicoulombs). She developed a generalized bilateral seizure, which lasted for 94 seconds monitored by double-channel electroencephalogram (EEG). Physically observed bilateral seizures were visible for 46 seconds. The postictal suppression index was not detected.

After five seconds, briefly before starting to regain consciousness, the patient developed a generalized tonic-clonic seizure. Intravenous diazepam 10 mg was administered twice, with temporary cessation in the seizure activity. The patient was treated with intravenous propofol and therefore intubated. She was transferred to the intensive care unit. Physical examination showed a haemodynamically stable patient, with signs of cerebral irritability; the pupil of her left eye was 1-2 mm more dilated compared to the right pupil, with no evidence of neck stiffness or petechiae. Bilateral ankle clonuses were discovered. Arterial blood gases revealed mild respiratory acidosis. Blood tests, chest X-ray, electrocardiography, head computed tomography angiography scan (CT), and magnetic resonance imaging (MRI) of the brain were normal. Lumbar puncture cell count was within normal limits, and there was no evidence of markers of autoimmune disease. Repeated EEGs showed generalized tonic-clonic epileptiform activity over both hemispheres.

First, after eight days, seizure was completely terminated by simultaneous drugs of propofol, midazolam, fosphenytoin, lacosamide, and brivaracetam under intubation. Barbiturate anaesthesia was not used for unknown reasons. In addition, she was treated with multiple agents: fentanyl, clonidine, methadone, prednisone, olanzapine, and haloperidol. The patient developed severe akathisia and postictal delirium postextubation. In order to prevent the patient from harming herself, physical fixation to the bed by the usage of leather belts around the waist, wrists, and arms was necessary. The patient was stabilized and returned to the inpatient psychiatric unit, with no evidence of depressive symptoms twelve days after the ECT treatment. She remained well with euthymic mood and was discharged to her parents' home five weeks later, with follow-up meetings in both the psychiatric and the neurological outpatient clinics. The patient reported subjective cognitive deficits lasting for two years and underwent municipality-based physical rehabilitation in that period. There were no objective neurological sequelae or complications—in particular no reappearance of seizure or depressive symptoms after the patient was discharged. The patient was prescribed treatment with clomipramine (serum clomipramine 120 nmol/L), 400 mg lacosamide, and 200 mg of brivaracetam. The antiepileptic drugs were discontinued after one year without reappearing seizures.

## 3. Discussion

Development of convulsive SE is a rare complication following ECT treatment. This condition lasted for more than a week in this patient, despite several attempts by pharmaceutical intervention. Several predictive factors for SE induced by ECT are described in the literature [9, 10]. Withdrawal of benzodiazepine and anticonvulsant agents increases the risk of status epilepticus [5], and lithium carbonate [7], antidepressants, and antipsychotics [5] may potentially lower the seizure threshold. Our patient was treated with lithium carbonate, which may have had an influence on the outcome. Moreover, the risk to develop SE increases, when repeated seizures are induced during the same treatment session [1].

Patient and family history of seizure is found to be a risk factor. The fact that the patient had a history of convulsive syncope may be considered a risk factor for SE. The history of minor traumatic subarachnoid haemorrhage may also contribute to the aetiology of the SE in this case.

The predictive factors, mentioned above, might have an impact on the development of convulsive SE; however, the actual aetiology in our case remains unclear.

This case emphasizes the importance of achieving a detailed review of the patient's medical history to detect possible risk factors that may contribute to SE following ECT. A combination of psychotropic medication should as well be considered carefully before initiating ECT.
